# The role of WDR76 protein in human diseases

**DOI:** 10.17305/bjbms.2020.5506

**Published:** 2021-10

**Authors:** Jie Yang, Fei Wang, Baoan Chen

**Affiliations:** 1 Department of Hematology and Oncology, Zhongda Hospital, School of Medicine, Southeast University, Nanjing, China

**Keywords:** WD40 repeat, WDR76, ubiquitination, human disease

## Abstract

The WD40 repeat (WDR) domain is one of the most abundant protein interaction domains in the human proteome. More than 360 protein interaction domains have been annotated thus far. The WDR domains mediate interactions with peptide regions of important interaction partners in a variety of biological processes. Proteins with the WDR domain which typically contains a seven-bladed β propeller, are continuously being discovered. They represent a large class of proteins that are likely to play important roles. WD40 repeat domain-containing protein 76 (WDR76) is a member of WDR domain-containing proteins. Although it remains poorly understood, it is potentially involved in DNA damage repair, apoptosis, cell cycle progression, and gene expression regulation. Ongoing research on WDR76 is increasing the knowledge regarding its basic functions and role in different pathophysiological. The study of WDR76 is challenging due to the complexity of its interactions with its partners. In the present review, we summarized the current knowledge regarding WDR76, its physiological functions, the close relationship with human diseases, and potential opportunities for target therapy.

## INTRODUCTION

With the research development with WD40 repeat (WDR) domain-containing proteins, play vital roles in diverse biological processes. Typically, the WDR domain contains 4-8 repeating sequence units of 40-60 amino acids ending with a tryptophan-aspartate (WD) dipeptide [1]. Thus far, it has been recognized as the fourth most abundant domain in the human proteome [1].WDR domain-containing proteins generally contain at least one such domain, and the commonest one contains seven repeats folded into a seven-bladed β propeller [2]. Owing to the complex structure of WDR domains, they often act as essential subunits of multiprotein complexes involved in a variety of cellular processes, including signaling transduction, DNA damage sensing and repair, the ubiquitin–proteasome system, cell growth and division, epigenetic regulation of gene expression and chromatin organization [3-6]. As a result, members of this WDR domain-containing protein family are characterized by unique functions [7].

WDR76 is a poorly characterized protein which is predicted to be a member of the WDR domain-containing superfamily [8]. It was predicted that the crystal structure of WDR76 includes a β propeller architecture containing seven WD40 blades, which determines its specific functions to a large extent. YDL156w/changed mutation rate 1 (YDL156w/CMR1), the homologue of WDR76, was first described in *Saccharomyces. cerevisiae*; it has exhibited histone affinity [9]. Moreover, it participates in diverse biological process, including UV-damage DNA binding[10], DNA metabolism [11], promotion of transcription [12], etc. The mouse homologue of WDR76 showed strong binding affinity for to H3K27 acetylation (H3K27ac) and H3 lysine 4 trimethylation (H3K4me3) in mouse embryonic stem cells [13]. Further research studies on the function of WDR76, have demonstrated that it is involved in multiple distinct pathophysiological processes, particularly in different diseases including tumorigenesis [14, 15]. Until now, WDR76 has shown to play a vital role in types of cancer, such as liver cancer[16] and colorectal cancer (CRC) [17].

According to the currently available results, WDR76 is identified as a hub protein. However, the current understanding of WDR76 compared to other members of WDR domain-containing superfamily is very limited. Hence, the present review summarized the recent progress on WDR76 research with the aim to better understand the WDR76 protein and eventually lead to potential clinical target therapy in different diseases.

### Structure of the WDR domain and WDR76

Generally, the WDR domain contains a conserved serine-histidine and WD motif and it can retain its β propeller fold due to its structural adaptability [18]. Each blade further comprises a four-stranded anti-parallel β-sheet [19, 20]. The WDR domain usually acts as a scaffold present in large multiprotein complexes [1]. Based on tis three-dimensional structure, the top, bottom, and side surfaces of the WDR domain can simultaneously act as interaction sites for various binding partners, including peptides, proteins, RNA and DNA [21]. For example, the WDR domain of DNA-damage-binding protein 1 (DDB1) binds to damaged DNA [22], while the WD repeat domain-containing protein 5 (WDR5) binds to methylated arginine [23].

The WDR domain, which is best characterized in *Saccharomyces.cerevisiae* for interactomes, is the fourth most abundant domain in the human proteome [1]. Because of the low sequence conservation and functional diversity of WDR domains, it is difficult to identify all the WDR domain-containing proteins. An algorithm termed WDSP was developed to detect a large number of WDR domain-containing proteins from different species [24, 25]. For most of those, further exploration is required to reveal their structures and functions. It was reported that the WDR domain is part of a larger class of β propeller domains, which do not have sequence similarity but exhibit structurally similar folds. These domains are involved in numerous potential complex functions [26, 27].

**FIGURE 1:**
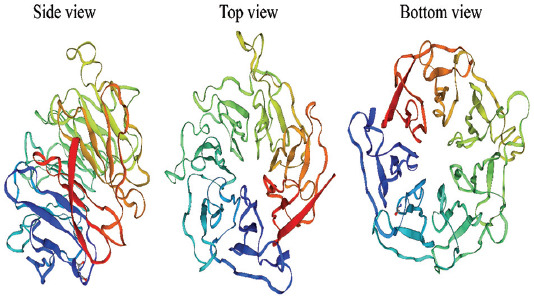
Three-dimensional predicated structure of WDR76 with web-based sever Swiss Model. Different color represents seven WD40 blades. WDR76: WDR domain-containing protein 76.

WDR76 is a poorly characterized member of the WDR domain-containing protein family. Recently, Dayebgadoh et al carried out a phylogenetic analysis on 17 different sequences of WDR76 homologues downloaded from the National Center for Biotechnology Information (NCBI) [8]. The results showed that human WDR76 was closer to its homologues in vertebrates versus non-vertebrates, and it was closet to WDR76 in *Macaca mulatta*. Moreover, they predicted the presence of a C-terminal WDR domain in all 17 homologues of WDR76 including human WDR76. Therefore, it appears that WDR76 is conserved in higher eukaryotes. Currently, the crystal structure of human WDR76 not been determined, Swiss Model [28], a web-based sever, was used to visualize the structure of WDR76 (Figure 1). The analysis confirmed that the three-dimensional structure of the WDR domain of WDR76 included a coiled β propeller architecture containing seven WD40 blades. WD1 (blue colored) was the first N-terminal WD40 blade and WD7 (red colored) was the last C-terminal WD40 blade. More recently, Mistry et al indicated that WDR76 protein is a member of the DDB1 and CUL4-associated factor (DCAF) subfamily by utilizing the search terms WD40, DDB1-binding/WD40 domain (DWD), CUL4-DDB1-associated WDR (CDW), DCAF, DDB1/2 and CUL4 in the available public databases and PubMed [29].

### Diverse interactions of WDR76

**FIGURE 2:**
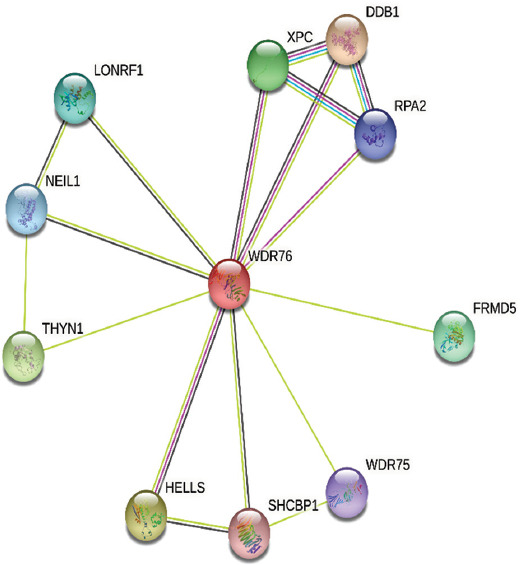
Analysis of WDR76 protein interaction network. WDR76: WDR domain-containing protein 76.

Similar to a large number of members of the WDR domain-containing protein family, WDR domain-containing proteins may present their specific functions through sequences outside the WDR domain. Thus far, limited research evidence suggested that WDR76 participates in several interaction processes, such as the protein-protein interaction (PPI) network showed in Figure 2 according to STRING database (https://string-db.org) [30]. However, according to the currently available research findings, thymocyte nuclear protein 1 (THYN1), WD40 repeat domain-containing protein 75 (WDR75), and FERM domain-containing protein 5 (FRMD5) have only been mentioned together with WDR76 in other organisms. XPC, LON peptidase N-terminal domain and ring finger 1 (LONRF1), nei like DNA glycosylase 1 (NEIL1), and SHC binding and spindle associated 1 (SHCBP1) are co-expressed with WDR76 in different organisms other than homo sapiens. Only the remaining proteins, including DDB1, helicase, lymphoid specific (HELLS), and replication protein A2 (RPA2) were identified in the following studies which are summarized in Table 1.

**TABLE 1:**
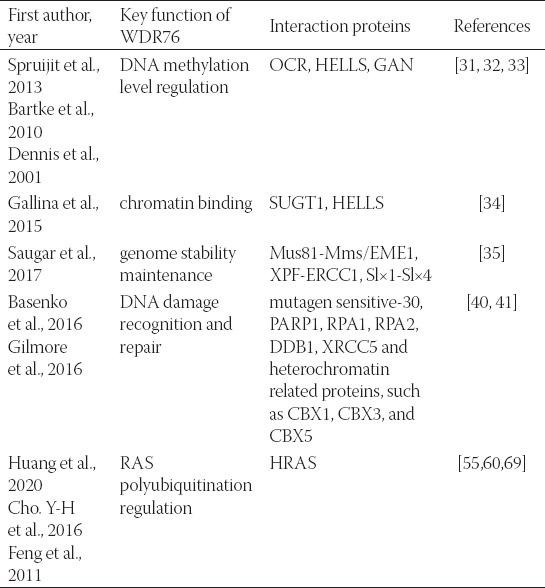
Key functions and interactions of the WDR76 protein

Spruijit et al found that WDR76 preferentially binds to 5-hydroxymethylcytosine (5hmC) than to cytosine (C). Purified WDR76 was treated as a green fluorescent protein (GFP) fusion protein and was found to interact with OCR, HELLS, and gigaxonin (GAN) [31]. OCR, Spindlin-1, is a protein that can bind H3K4me3 [32]. Mouse protein hells is implicated in the regulation of DNA methylation [33]. Gallina et al demonstrated that WDR76, the closest orthologue of Cmr1 in higher eukaryotes, interacted with SGT1 homologues MIS12 kinetochore complex assembly cochaperone (SUGT1) and HELLS, two chromatin-related proteins [34]. Next, they utilized a GFP-WDR76-expressing plasmid and then found it localized to nuclear foci. Subsequently, the participation of WDR76 in specific endonucleases was also reported [35]. Structure-specific endonucleases play an essential role in the maintenance of genome stability, as they mediate several biological processes including DNA replication, DNA repair, and DNA recombination [36-38]. Saugar et al showed that endonuclease Mus81-Mms/ essential meiotic structure-specific endonuclease 1 (Mus81-Mms/EME1) relocalized to subnuclear foci following DNA damage, together with other endonucleases Rad1-Ras10 (XPF-ERCC1) and Slx1-Slx4 [35]. The recruitment was caused by Cmr1/WDR76, a protein involved in preserving genome stability; this process depended on the E2-ubiquitin-conjugating enzyme Rad6 and the E3-ubiquitin ligase Bre1 [39]. Another study showed that the level of mutagen sensitive-30 (*MUS-30*), a nuclear protein, increased following DNA damage [40]. Deletion of WDR76 rescued the DNA damage-hypersensitivity of D*mus-30* strains. In addition, the *MUS-30-WDR76* interaction was shown to be functionally important in DNA damage repair.

Gilmore et al focused on the interaction network of WDR76 and its recruitment to laser induced DNA damage [41]. The results showed that WDR76 was specifically associated with DNA damage response proteins, such as poly(ADP-ribose) polymerase 1 (PARP1), poly(ADP-ribose) polymerase 1 (RPA1), RPA2, DDB1, and poly(ADP-ribose) polymerase 1 (XRCC5), as well as heterochromatin related proteins, such as chromobox 1 (CBX1), chromobox 3 (CBX3), and chromobox 5 (CBX5). In addition, it was shown that WDR76 recruited immediately after laser induced DNA damage and in markedly higher level than other heterochromatin related proteins, such as CBX1 and CBX5. Subsequently, the investigator of that study conducted a more systematic and in-depth study on the WDR interaction network. They expressed SNAP-tagged full length WDR76 (1-626) and two WDR76 deletion mutants in HEK293T cells including the C-terminal deletion of WDR76 (*WDR76Δ*) and the N-terminal deletion of WDR76 (*WDR76Δ’*)[8]. Furtherinvestigation on tineractions revealed that full-length WDR76 interacted with the chaperonin-containing protein 1 (TCP1) or TriC-TCP-1 Ring (CCT) complex, histones, the nicotinamide adenine dinucleotide-dependent deacetylase sirtuin 1 (SIRT1), GAN, DNA- dependent protein kinase-Ku (DNA-PK-Ku) and PARP1. However, *WDR76Δ* lost the ability to interact with the CCT complex, and *WDR76Δ’* showed a significance decrease of WDR76 interaction with SIRT1, GAN, histones, protein kinase, DNA-activated, catalytic subunit (PRKDC), XRCC5, XRCC6, and PARP1. These results indicated that WDR76 interacted with partners via different regions instead of the WDR domain only.

The currently available evidence underlines the challenges faced by researchers in studying the WDR76 protein due to its complex interactions covering a diverse series of biological pathways. Proteins involved in the PPI map require further study to clarify the role of WDR76.

### Functions of WDR76 in diseases

The CUL4-DDB1- regulator of cullins 1 (CUL4-DDB1-ROC1) complex belongs to the family culling-containing ubiquitin E3 ligases [42]. Previous studies showed that the CUL4-DDB1-ROC1 complex participates in several biological processes, including cell-cycle progression, replication and DNA damage response [43-45]. DCAF proteins are involved in substrate recognition and protein recruitment for ubiquitination, which confer specificity to cullin-4 RING ubiquitin ligase (CRL4) [46, 47]. Protein ubiquitination is an essential modification process that exactly regulates a broad range of cellular and developmental processes [48-50]. A number of WDR domain-containing proteins function as DCAF proteins to determine the specific substrates[51]; these proteins are obviously related to several human diseases [52-54]. In previous research, WDR76 was identified as a member of the DCAF subfamily involved in histone methylation; an increasing number of studies on its unique role in different diseases have also been conducted.

Through proteomic analysis, Huang et al identified two potential WDR domain-containing proteins, namely ­CUL4-associated factor 8 (DCAF8) and WDR76, as substrate receptors of CRL4 family E3 for ubiquitination [55]. Lymphoid-specific helicase (LSH), a member of the SNF2 family chromatin remodeling ATPases, cooperates with histone and/or DNA modifiers to suppress the expression of ferroptosis genes involved in the occurrence and development of many tumor diseases [56]. DCAF8 was further confirmed to incorporate into a functional CRL4^DCAF8^ E3 ligase complex to catalyze LSH polyubiquitination. In contrast, WDR76 suppressed DCAF8-targeted LSH ubiquitination and proteasomal degradation through competitive inhibition of holo-CUL4-DDB1-DCAF8-LSH complex formation instead of direct interaction with E3 complex components. Molecular action of WDR76 in suppressing LSH incorporation into a functional complex corresponded to the distinct ferroptosis in cancer cells. The opposing regulation of LSH stability by WDR76 and DCAF8 decreased DNA oxidation and overproduction of reactive oxygen species (ROS), which was a crucial process in epigenetic regulation of ferroptosis. Transcriptomic profiling further revealed that the DCAF8/WDR76/LSH axis may be a critical epigenetic modulator of ferroptosis.

RAS proteins (H, K, and NRAS) are small guanosine triphosphatases (GTPases), which play essential roles in various pathophysiological regulatory, including cell proliferation, transformation, and development [57]. The RAS mutations are common in many types of human cancers [58,59]. Overexpression of RAS protein can also affect the occurrence of cancer, including hepatocellular carcinomas (HCC) and is associated with poor prognosis in patients [60]. Stabilization of RAS proteins participates in the activation of downstream signaling pathways associated with tumorigenesis [61-64]. Jeong et al used purified glutathione-S-transferase (GST)-fused HRAS protein (GST-HRAS) to determined HRAS-binding partner proteins in tissues of HCC tumors expressing high level of RAS compared with normal liver tissues [16]. They successfully identified WDR76, a CUL4-DDB1 ubiquitin E3 ligase interacting protein, as a tumor suppressor candidate. In vivo and in vitro studies demonstrated that cytoplasmic WDR76 directly mediated RAS polyubiquitination degradation through binding to HRAS, resulting in inhibition of proliferation, transformation, and the invasive ability of liver cancer cells. Tissue section staining also showed markedly higher intensities of RAS staining in HCC tissues compared with normal tissue, and negative correlation with the staining intensities of WDR76 in tumor tissues. Targeting the increase of WD76 expression level in liver tissue may prevent further development of HCC.

Then another related study to explore the function of WDR76 regulating HRAS protein stability in high-fat diet (HFD)-induced obesity and hepatic steatosis [65]. The results showed that WDR76 mediated adipocyte differentiation of 3T3-L1 cells. In mice, overexpression and knockdown of WDR76 decreased and increased the level of HRAS protein, respectively. WDR76 mediated HRAS degradation via polyubiquitination-dependent proteasomal degradation in 3T3-L1 cells [66]. As hepatic steatosis is one of the factors that can progress to HCC; hence this study provided new insights into the potential targeted treatment of hepatic steatosis through regulation of WDR76 expression to prevent the transformation of liver cells into cancer cells.

CRC is a stem cell disease that occurs when the intestinal stem cells (ISCs) evade regulation and give rise to cancer stem cells (CSCs) [67, 68]. Wnt/β-catenin signaling pathway maintains the self-renewal of normal ISCs [69] and genetic alterations in this signal pathway can result in CRC tumor progression [70]. Wnt/β-catenin signaling is regulated via the MAPK/ERK and PI3K/AKT pathways, which are the major effector pathways downstream of RAS [71]. *Adenomatous polyposis coli (APC)* mutation is common in patients with CRC, with an approximately 90% occurrence rate. Through aberrant activation of the Wnt/β-catenin signaling pathway. K-RAS mutation with 40-50% occurrence rate in CRCs cannot result in CSC only by itself [72]. However, simultaneous occurrence of the two aforementioned mutations can lead to CSC activation, tumorigenesis, and metastasis [73]. WDR76 has been identified as an interactive partner of HRAS in HCC and destabilizes all three major RAS isoforms [16]. Researchers hypothesized that WDR76 destabilized RAS through Wnt/β-catenin signaling pathway and further acted as a tumor suppressor to inhibit CSC activation in CRC [17]. In mice model with loss of *Wdr76*, the protein levels of RAS were increased by activating the Wnt/β-catenin pathway, thereby inducing significant crypt lengthening and hyper-proliferation. *Apc* mutation and *Wdr76* knockout mice exhibited increases in both the number and the size of tumors in small intestine. Besides, the study demonstrated that WDR76 reduced RAS level through polyubiquitination-dependent proteasomal degradation with *APC*-mutant D-WT and D-MT cells, which are isogenic except for harboring wild-type and mutant *K-RAS*, respectively. On the contrary, loss of WDR76 in CRC increased the level of RAS through the activation of the Wnt/β-catenin signaling pathway. This result suggests that destabilization of RAS by WDR76 is a potential treatment method targeting CRC involving CSC activation.

Alzheimer’s disease (AD) is a common disorder, and its incidence increases significantly with age [74]. However, the currently available treatments are not effective. Raghavan NS with his colleagues used whole-exome sequencing in 20,197 individuals from different centers to determine rare variants in AD [75]. A collapsing method was used to identify disease associated genes. WDR76 was screened out as the second top-ranked gene and its variants were linked to AD. Nevertheless, the mechanism through which WDR76 functions in this setting has not been elucidated. Further experiments are warranted to address this problem.

## CONCLUSION

Great progress has been achieved on research regarding the WDR domain-containing superfamily. An increasing number of members of this family are found to be involved in multiple biological processes of various diseases. WDR76 is considered a hub protein with a diverse array of more than 100 interactions were found. The results of relevant studies have indicated that it is closely linked to a variety of diseases, including different types of cancer. Based on the studies available, WDR76 is identified as a novel tumor suppressor and RAS activity can be controlled via regulating protein stability which further affect the tumorigenesis and metastasis in different cancer diseases. The process of RAS protein stability regulation is complex and involves multiple signaling pathways. Therefore, regulation of RAS at the level of protein stability by WDR76, which mediating polyubiquitination-dependent degradation of RAS, may be a new therapeutic strategy. However, further research is warranted to elucidate the complex mechanisms through which WDR76 functions. This approach may clarify related pathophysiological processes, potentially leading to the discovery of effective target treatments.
